# Supplementary material for: Evaluation of the introduction of a healthy food and drink policy in 13 community recreation centres on the healthiness and nutrient content of customer purchases and business outcomes: An observational study
Source: PLoS One. 2023 Jul 19;18(7):e0288719. doi: 10.1371/journal.pone.0288719 (PMC10355379; doi:10.1371/journal.pone.0288719)
Supplement: S1 Table — ¥ Pooled meta-analysis estimates using a random effects REML model. # % sales outcomes based on total volume sold. Mean centre marginal means estimated at each study period. I2 estimate of the percentage of the between study variability. & Cochran’s Q test for heterogeneity: p<0.001 for all outcomes. CI confidence interval. (DOCX) [file pone.0288719.s001.docx]

| **Outcomes ^#^** | **Pre-implementation** | **Post-implementation** | **Difference (Post – Pre)** | | | **I^2^** **Test for** | |
| --- | --- | --- | --- | --- | --- | --- | --- |
|  | Mean (95% CI)^¥^ | Mean (95% CI) ^¥^ | Overall difference (95% CI) ^¥^ | p-value | | **Heterogeneity**^&^ | |
| Healthiness |  |  |  |  |  |  |  |
| Food ‘Red’ (%) | 84 (76 to 91) | 67 (60 to 75) | -16 (-23 to -9.9) | <0.001 |  | 94% |  |
| ‘Amber’ (%) | 12 (6.7 to 17) | 26 (20 to 32) | 14 (9.1 to 19) | <0.001 |  | 94% |  |
| ‘Green’ (%) | 4.2 (0.43 to 7.9) | 6.2 (1.9 to 10) | 2.0 (-1.9 to 6.0) | 0.308 |  | 100% |  |
| Drinks ‘Red’ (%) | 51 (45 to 57) | 8.5 (5.2 to 12) | -41 (-48 to -34) | <0.001 |  | 93% |  |
| ‘Amber’ (%) | 12 (6.9 to 16) | 23 (16 to 30) | 11 (4.3 to 18) | 0.002 |  | 97% |  |
| ‘Green’ (%) | 38 (33 to 43) | 67 (61 to 74) | 30 (22 to 37) | <0.001 |  | 95% |  |
