# Supplementary figures and images for: Evaluation of the introduction of a healthy food and drink policy in 13 community recreation centres on the healthiness and nutrient content of customer purchases and business outcomes: An observational study
Source: PLoS One. 2023 Jul 19;18(7):e0288719. doi: 10.1371/journal.pone.0288719 (PMC10355379; doi:10.1371/journal.pone.0288719)

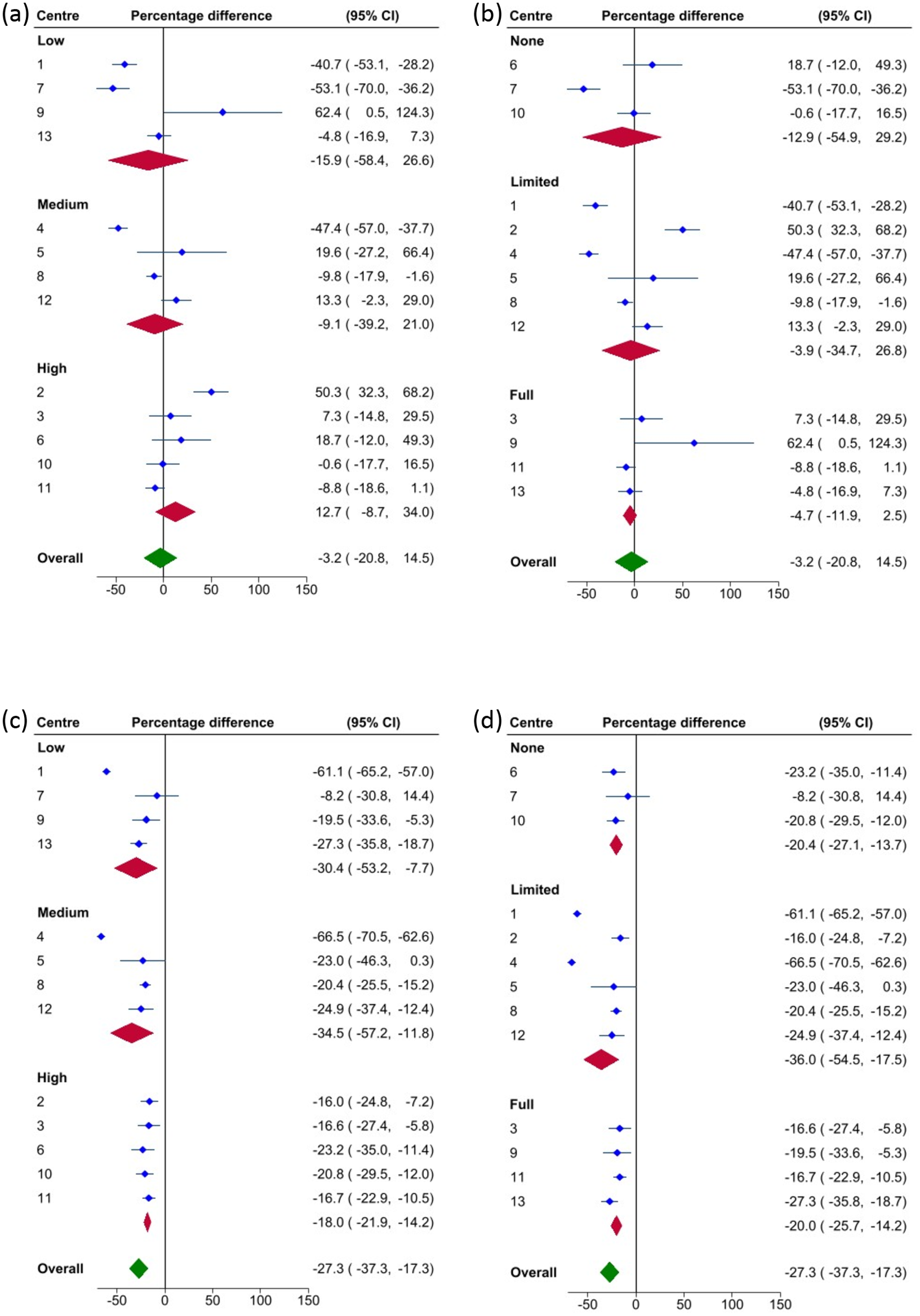

Supplement: S1 Fig — a) Food by SEP, b) Food by type of preparation facilities, c) Drink by SEP and d) Drink by type of preparation facilities. # Relative percentage difference = (post–pre-implementation) / pre-implementation× 100. Overall effect estimated using pooled meta-analysis estimates using a random effects REML model. CI: confidence interval. SEP socioeconomic position of centre measured using the centre postcode and the Australian Bureau of Statistics Socio-Economic Indexes for Areas (SEIFA), Index of Relative Socio-Economic Advantage and Disadvantage (IRSAD) with percentiles for the state classified as being high SEP (IRSAD percentile >65, relative lack of disadvantage and greater advantage), medium (IRSAD percentile 34–65), and low (IRSAD percentile < 34, relative greater disadvantage and a lack of advantage). (TIF) [file pone.0288719.s002.tif]

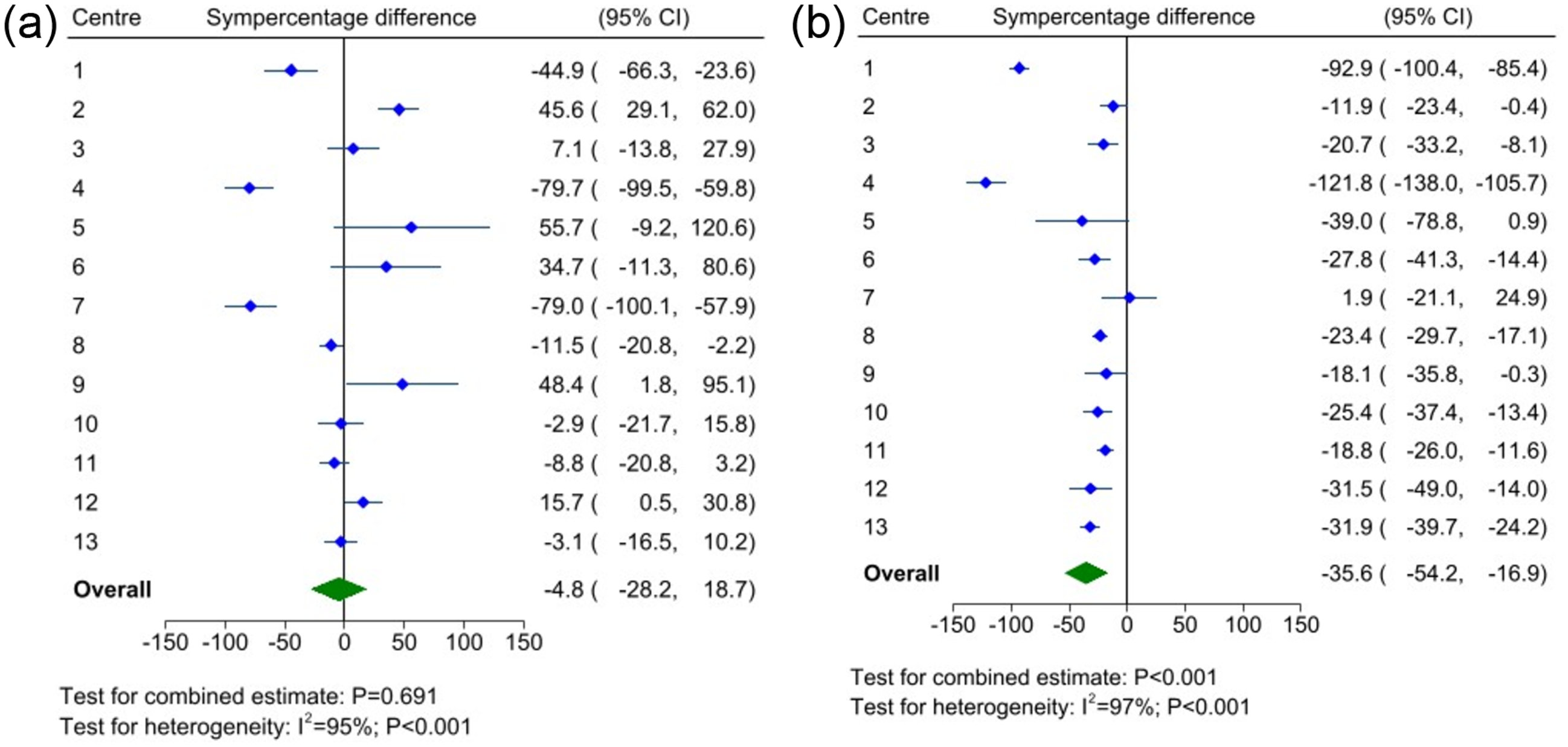

Supplement: S2 Fig — a) Food and b) Drink. # percentage difference on the 100 log(e) scale. Overall effect estimated using a random effects REML model. CI: confidence interval. (TIF) [file pone.0288719.s003.tif]

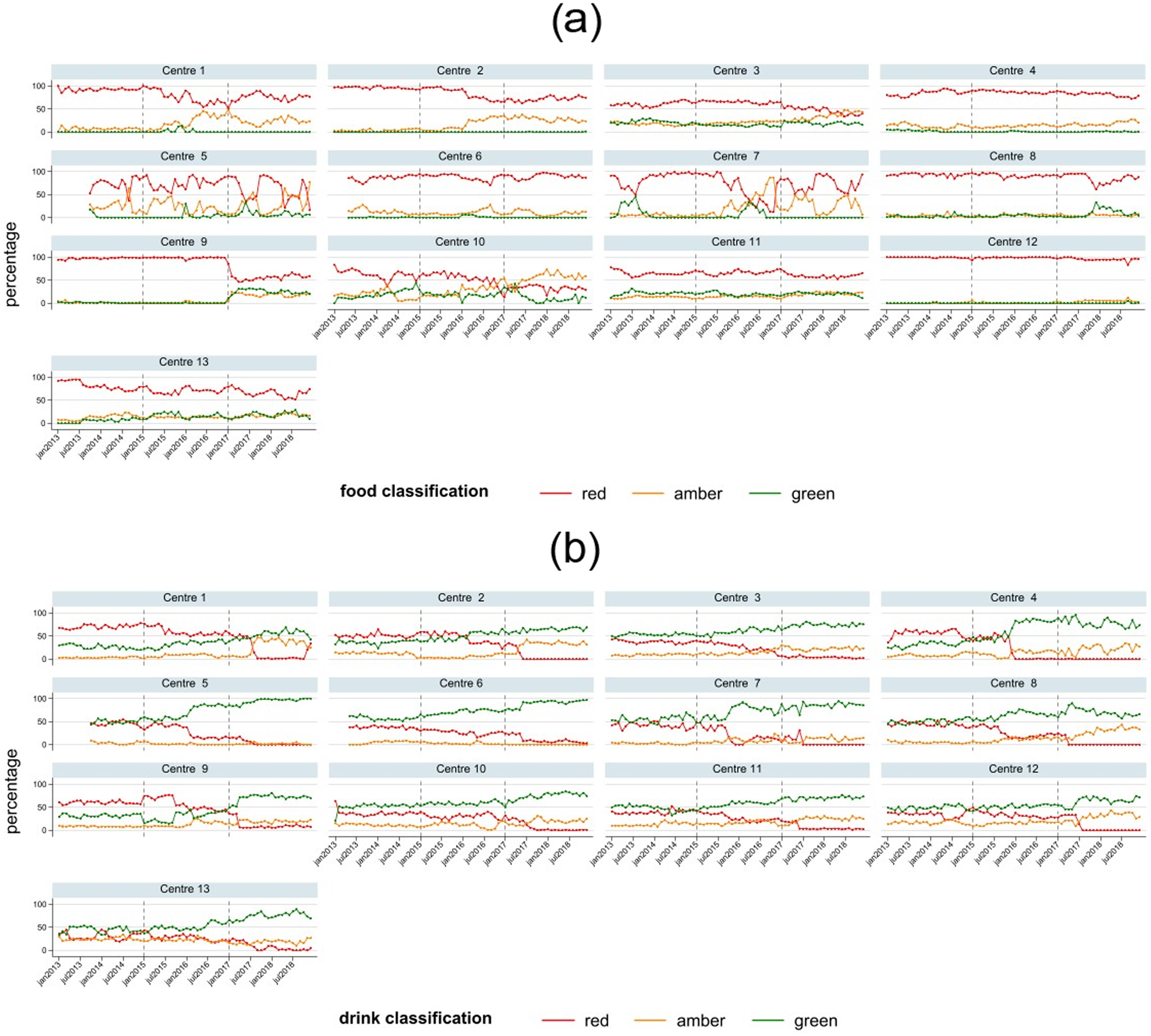

Supplement: S3 Fig — a) Food. # Dashed lines denote the date initiative started (1 January 2015) and date initiative was to be fully implemented (1 January 2017). b) Drink. # Dashed lines denote the date initiative started (1 January 2015) and date initiative was to be fully implemented (1 January 2017). (TIF) [file pone.0288719.s004.tif]

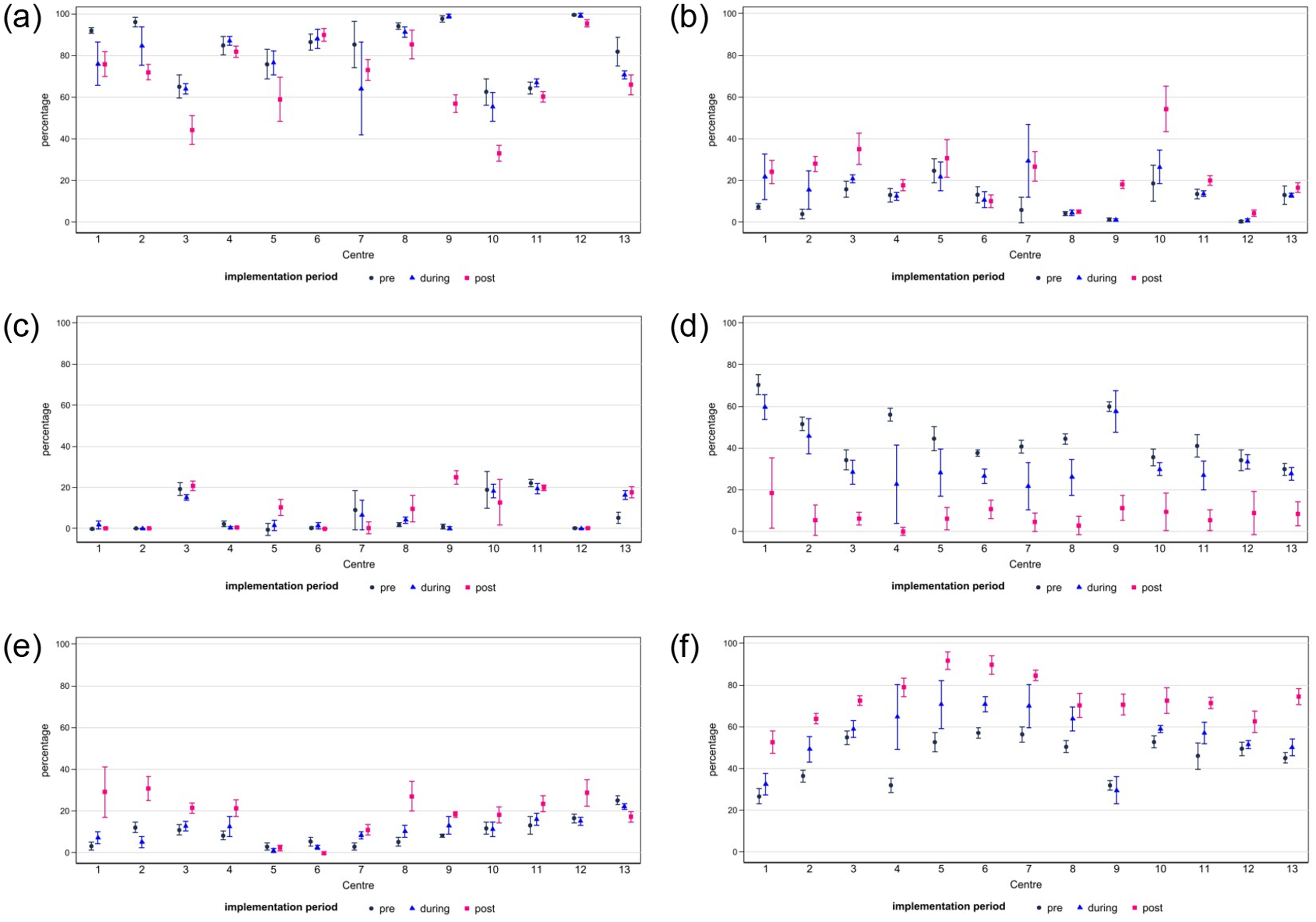

Supplement: S4 Fig — FOOD: a) ‘Red’ food, b) ‘Amber’ food, c) ‘Green’ food. DRINK: d) ‘Red’ drink, e) ‘Amber’ drink, f) ‘Green’ drink. # Marginal means and 95% confidence intervals (CI) for each centre were estimated from a linear model with Newey-West standard errors to accommodate for serial autocorrelation (lag 3) and adjusting for calendar month and monthly attendance. (TIF) [file pone.0288719.s005.tif]

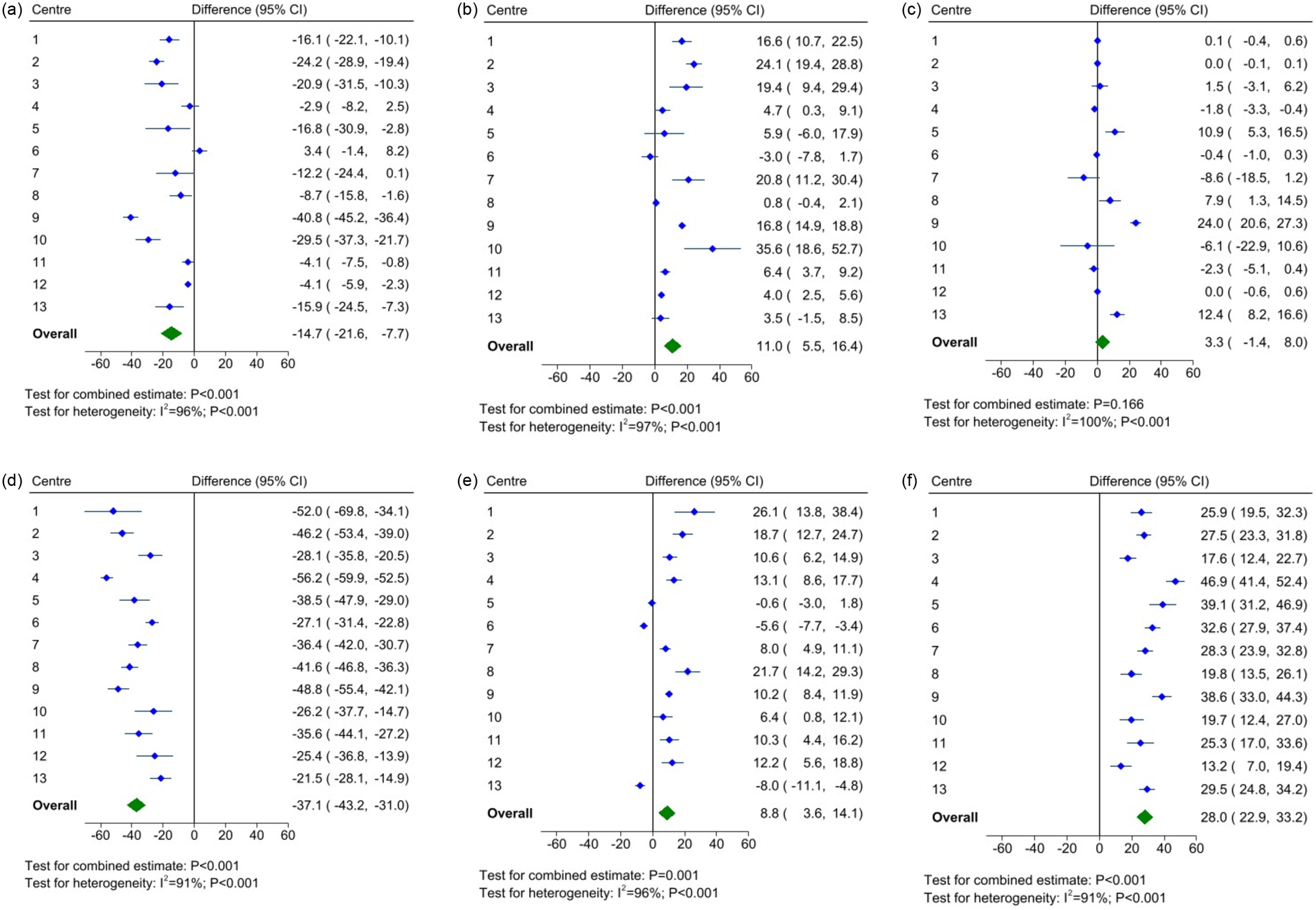

Supplement: S5 Fig — FOOD: a) ‘Red’ food, b) ‘Amber’ food, c) ‘Green’ food. DRINK: d) ‘Red’ drink, e) ‘Amber’ drink, and f) ‘Green’ drink. # Percentage difference = (post–pre-intervention) × 100. Overall effect estimated using pooled meta-analysis estimates using a random effects REML model. CI: confidence interval. (TIF) [file pone.0288719.s006.tif]

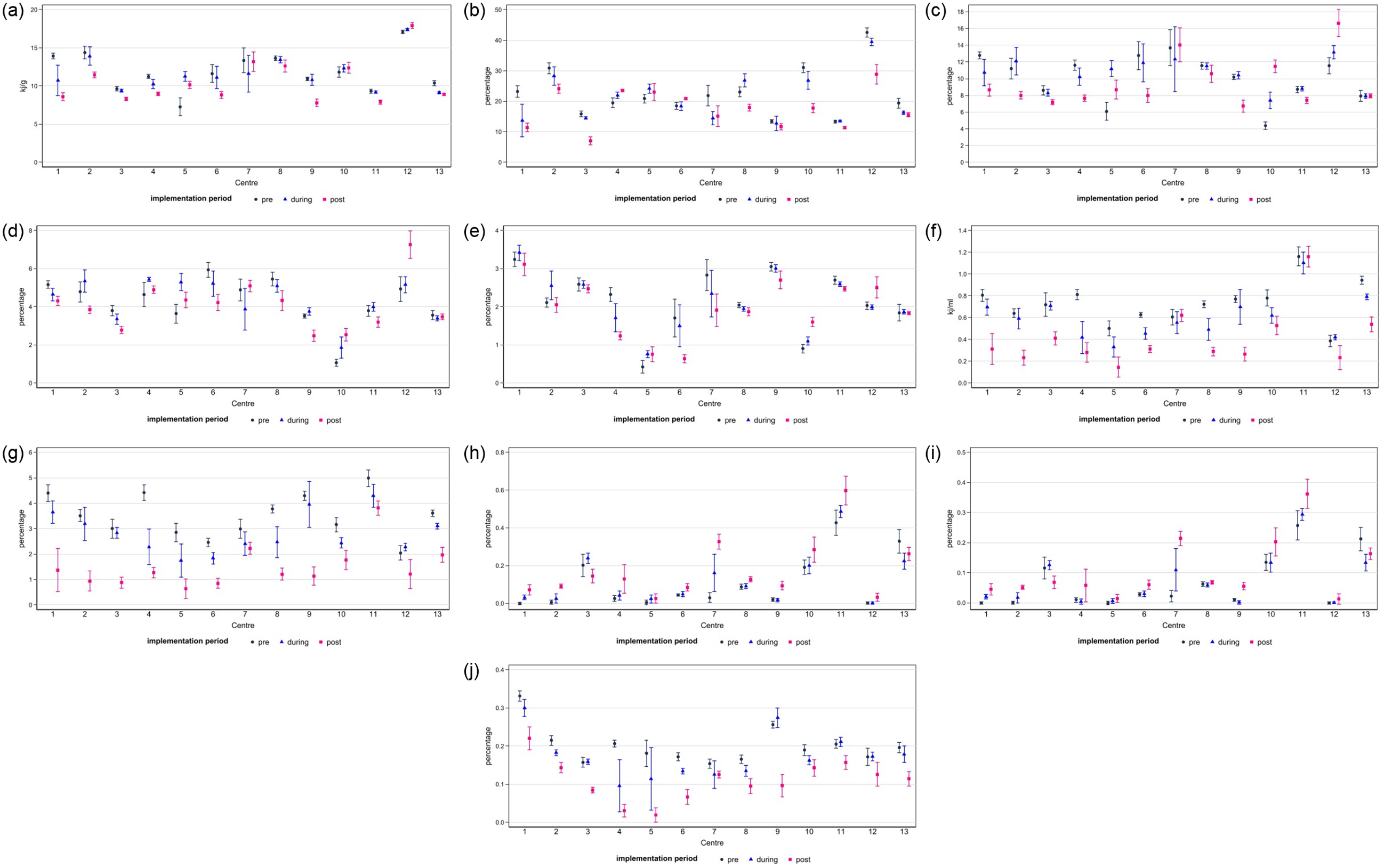

Supplement: S6 Fig — FOOD: a) Energy density (kJ/g), b) Percentage volume of sugar, c) Percentage volume of total fat, d) Percentage volume of saturated fat, and e) Percentage volume of salt. DRINK: f) Energy density (kJ/g), g) Percentage volume of sugar, h) Percentage volume of total fat, i) Percentage volume of saturated fat, and j) Percentage volume of salt. # Marginal means and 95% confidence intervals (CI) for each centre were estimated from a linear model with Newey-West standard errors to accommodate for serial autocorrelation (lag 3) and adjusting for calendar month and monthly attendance. (TIF) [file pone.0288719.s007.tif]

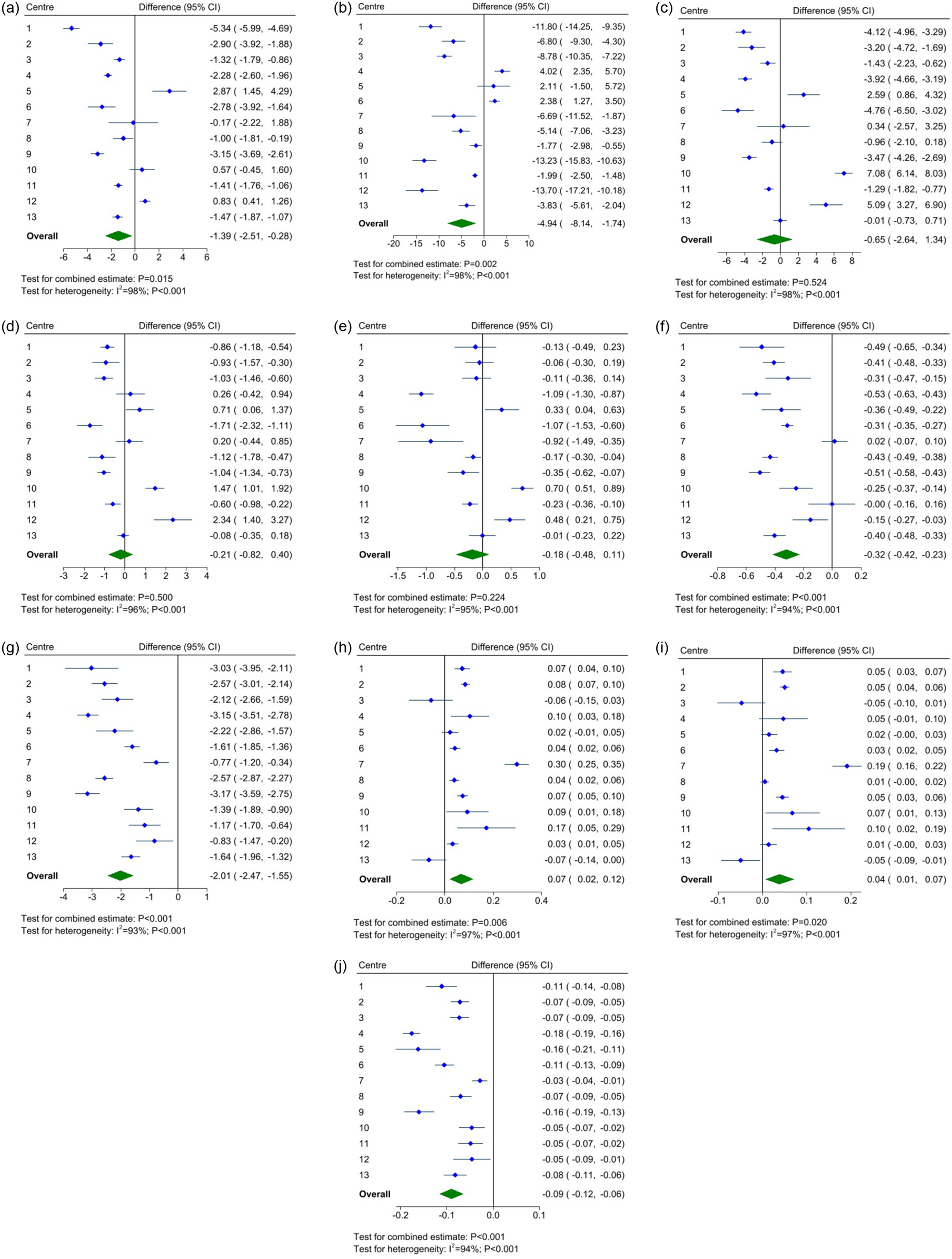

Supplement: S7 Fig — FOOD: a) Energy density (kJ/g), b) Percentage volume of sugar, c) Percentage volume of total fat, d) Percentage volume of saturated fat, e) Percentage volume of salt. DRINK: f) Energy density (kJ/g), g) Percentage volume of sugar, h) Percentage volume of total fat, i) Percentage volume of saturated fat, j) Percentage volume of salt. # Difference = (post–pre-intervention). Overall effect estimated using pooled meta-analysis estimates using a random effects REML model. CI: confidence interval. (TIF) [file pone.0288719.s008.tif]
